# The Effect of Religiosity on Pro-environmental Behavior Based on the Theory of Planned Behavior: A Cross-Sectional Study Among Iranian Rural Female Facilitators

**DOI:** 10.3389/fpsyg.2022.745019

**Published:** 2022-02-25

**Authors:** Saeid Karimi, Genovaitė Liobikienė, Fatemeh Alitavakoli

**Affiliations:** ^1^Department of Agricultural Extension and Education, Faculty of Agriculture, Bu-Ali Sina University, Hamedan, Iran; ^2^Department of Applied Economics, Finance and Accounting, Vytautas Magnus University Agriculture Academy, Akademija, Lithuania

**Keywords:** religiosity, theory of planned behavior, pro-environmental behaviors, rural female facilitators, Iran

## Abstract

Religiosity is one of the most prominent and extensive social factors influencing one’s behavior; however, there is a lack of research analyzing the religiosity impact on pro-environmental behavior, particularly for women in rural areas. To narrow the research gap, this study established a theoretical research model by incorporating religiosity into the Theory of Planned Behavior to explore factors affecting rural female facilitators’ pro-environmental behavior. The extended Theory of Planned Behavior model was consequently tested by empirical data collected from 110 rural female facilitators in Qom Province in the center of Iran. The results of structural equation modeling indicated that subjective norms and environmental attitude were positively and significantly related to pro-environmental intentions. In addition, pro-environmental intentions and perceived behavioral control were found to be significantly associated with pro-environmental behaviors. The results revealed that there was a direct and indirect relationship between religiosity and pro-environmental behaviors via perceived behavioral control. In addition, there was an indirect relationship between religiosity and pro-environmental intentions via subjective norms and environmental attitudes. Therefore, this study revealed that religiosity as social pressure plays an important role in determining pro-environmental intentions and behaviors among rural female facilitators in Iran. Thus, in order to promote pro-environmental behavior, the religiosity aspects should be considered and people should be stimulated to act in a more environmentally friendly mode via religious prism.

## Introduction

Today, the world faces several environmental problems such as air pollution, water scarcity, and global warming, which are jeopardizing planet Earth’s welfare and security. Unfortunately, Iran is in a critical environmental condition similar to many other countries, and this situation is growing worse. Regarding the Environmental Performance Index (EPI), Iran has 105th place among the 180 countries of the world, which indicates the inadequacy of actions taken for the sustainable protection and enhancement of the environment (Hsu and Zomer, 2016). The increasing growth of environmental problems and their harmful effects on Iran and other countries of the world reflect the necessity of finding immediate solutions. Since most environmental problems of today are mainly the results of human activities and actions, the implementation of the possible solutions to these growing problems calls for a behavioral change and complete people participation (Onel and Mukherjee, 2015). In other words, many environmental problems are directly linked to human behavior (Steg and Vlek, 2009; Hirsch, 2010), hence their solutions depend on changing this behavior and finding behavioral solutions (Hirsch, 2010; Ramkissoon et al., 2013; Steg et al., 2014). Therefore, the identification and analysis of the determinants of people’s pro-environmental behaviors are substantially important (Mancha and Yoder, 2015; Bergek and Mignon, 2017; Ramkissoon et al., 2018; Karimi, 2019; Karimi et al., 2021b). Researchers have explored various external, personal, psychological, and social factors (Gifford and Nilsson, 2014; Karimi, 2019; Kumar, 2019; Wang et al., 2019; Karimi and Saghaleini, 2021b), but an important factor, which is religiosity, has been scarcely analyzed (Ghazali et al., 2018).

### Why Religiosity

Religiosity is among the most prominent and extensive social institutions involved in the formation of almost any culture and society (Ives and Kidwell, 2019). According to a report by the Pew Research Center, 84% of the world’s population relies on one of the known religions (Pew Research Centre, 2017). Another estimate also suggests that almost 68% of the world’s population acknowledges the substantial role of religion in human daily life (Diener et al., 2011). Religiosity creates social norms, guides personal behaviors, and forms the basis for the social structures, ethics, and laws (Cohen, 2009). As regards to the environment, religion and religiosity could be expected to determine the individuals’ pro-environmental behaviors (PEBs), environmental concern, and attitude (Greeley, 1993; Stern et al., 1999; Bhuian and Sharma, 2017; Hwang, 2018). Religiosity, which is defined as the belief in the existence of God and the set of divine guidelines for human behavior and worldly actions (McDaniel and Burnett, 1990), can be a major source of environmental ethics (Rice, 2006; Vitell, 2009).

The effects of religiosity on women’s environmental behavior, especially rural female facilitators’ behaviors in one of the most religious Iranian cities (i.e., Qom), is significantly important. Rural women, especially rural female facilitators, are among the most important active rural groups that can significantly contribute to the attainment of sustainable development goals, in general, and environmental sustainability, in particular, in rural environments. In rural areas, women benefit from the environment either directly or indirectly. These women participate in many agricultural and rural activities and can play a determining role in cultural promotion and protection of the environment and natural resources both indoors and outdoors.

Therefore, this study makes two significant contributions to the literature. Firstly, it is among the very few studies that fully used the TPB framework to explain the pro-environmental behavior of rural female facilitators in a developing country and confirmed its application. Secondly, this study increased our understanding of the important role of religiosity in explaining environmental intentions and behaviors in an Islamic society. A literature review also revealed that the majority of the previous studies analyzed the relationship between these two constructs in western countries, which are mainly protestant and catholic countries and few studies have been conducted on the Islamic culture (Siyavooshi et al., 2019). Overall, this study provides novel empirical evidence and insights into the impact of religiosity on rural female facilitators’ pro-environmental behavior within the context of a developing country. This will lead to a better understanding of this area of study.

### Strategic Question

Following other researchers’ recommendations (Awan, 2011), the main question of this paper, which is raised, is whether there is a significant relationship between the level of religiosity of rural female facilitators in Qom Province and their PEBs. Given the importance of this issue, the present study attempts to integrate religiosity into the well-known theory of planned behavior (TPB: Ajzen, 1991) to study the association between religiosity and PEBs of rural female facilitators in Qom Province, Iran.

## Theoretical Framework and Hypotheses

### Pro-environmental Behavior and Theory of Planned Behavior

Pro-environmental behavior refers to a behavior that inflicts the minimum damage on the environment or even benefits the environment (Steg and Vlek, 2009). In other words, PEB is defined as the adoption of behaviors by individuals to promote environmental sustainability (Ramkissoon, 2020) and can contribute to sustainability promotion in the public sphere as organizations and companies (Awan et al., 2020; Kautish et al., 2021). To encourage people to perform PEBs such as reducing consumption of energy and resources, recycling, and reducing wastes and training them in these areas is particularly important (Dhir et al., 2021). Within the past four decades, numerous researchers have attempted to answer the following fundamental questions: Why do people show PEBs and what are the main barriers to PEBs (Kollmuss and Agyeman, 2002). Numerous theoretical frameworks such as Norm-Activation Theory (NAT: Schwartz, 1977), the Theory of Planned Behavior (TPB: Ajzen, 1991), and Value-belief-norms theory (VBN: Stern et al., 1999) have been developed to answer these questions. From the viewpoint of social psychology, the TPB offers a logical and appropriate framework to explain and predict behaviors and it has been widely adopted in different researches (Chin et al., 2016; Wang et al., 2019; Karimi et al., 2021a). The meta-analysis conducted by Overstreet et al. (2013) indicated that the theory of planned behavior (TPB) strongly predicts and explains intentions and behaviors. Moreover, the TPB has been applied to explain PEBs in different domains, including the workplace (Wesselink et al., 2017), energy conservation (Park and Kwon, 2017; Obaidellah et al., 2019), waste recycling (Oyekale, 2018; Kumar, 2019), meat consumption (Çoker and van der Linden, 2020), transportation usage (Cai et al., 2019; Shalender and Sharma, 2021), and environmental activism (Fielding et al., 2008). Many such studies have demonstrated that the TPB is suitable for explaining pro-environmental intentions and behaviors (Wang et al., 2019). However, this theory is less applied to analyze the Iran case, particularly none of the previous research focused on women in rural areas. Furthermore, in this paper, the extended TPB was suggested.

Referring to the classical definition of TPB, the behavioral intention of the individual is influenced by three motivational factors, namely attitude, subjective norms and perceived behavioral control (PBC), and behavioral intention eventually leads to actual behavior (Ajzen, 1991). PBC directly and positively affects actual behavior. Attitude toward a behavior is the degree of one’s favorable or unfavorable evaluation of the behavior in question. Positive evaluation of behavior and its outcomes increase the likelihood of engaging in the behavior (Ajzen, 1991). Generally, the more individual holds s positive attitude toward a behavior, the more likely he will intend to perform that behavior. Several studies, such as Tan et al. (2017), Khan et al. (2019), and Rezaei et al. (2019) have noted the importance of attitude in predicting an individual’s pro-environmental intentions in various contexts. For example, Rezaei et al. (2019) reported that attitude had a significant effect on Iranian farmers’ intention to use integrated pest management.

Subjective norms are the second important variable to affect an individual’s behavior intentions. Subjective norms refer to the individual’s perceived social pressure from others who are important to him that thinks he should or should not perform the behavior. The higher subjective norm individual perceived, the more likely to perform a behavior. This is also suitable for pro-environmental behaviors. If an individual realizes that most important people think he should perform pro-environmental behaviors, he will perceive pressures and intend to perform pro-environmental behaviors. A majority of the studies applying the TPB found subjective norms a significant determinant of pro-environmental intentions (e.g., Khan et al., 2019; Wang et al., 2019; Si et al., 2020). Subjective norms have been proved to be major predictors of the recycling intentions of households in Pakistan (Khan et al., 2019). PBC is another important variable affects an individual’s behavioral intentions and it defines as an individual’s perceived ease or difficulty in performing a specific behavior (Ajzen, 1991, 2005). This ease or difficulty assessment is a vital determinant in the actor’s decision to engage in a given behavior (Ajzen, 1991). More precisely, PBC measures an individual’s degree of having the opportunity and ability to perform a behavior. If individuals have a higher degree of control over themselves, they will have a stronger intention to perform a particular behavior. It has been consistently demonstrated in relation to pro-environmental behaviors that PBC can have a strong influence on various pro-environmental intentions and behaviors (Greaves et al., 2013; Wang et al., 2019; Si et al., 2020). For example, Si et al. (2020) found that PBC is the most important factors driving users’ sustainable usage intention and behavior in China. Besides, meta-analytic studies on various PEBs suggest that attitude, PBC, and subjective norms are strong predictors of pro-environmental intentions and behaviors (Bamberg and Möser, 2007; Klöckner, 2013; Maki and Rothman, 2017).

According to the TPB, the proximal determinants of behavior are intention to engage in that behavior and perceptions of control over that behavior. Intentions represent a person’s motivation in the sense of their conscious plan or decision to exert effort to perform the behavior (Ajzen, 1991). Previous studies indicated that pro-environmental intentions and PBC are significantly related to pro-environmental behaviors (De Leeuw et al., 2015; Wang et al., 2019; Si et al., 2020). For example, a previous survey conducted by Wang et al. (2019) revealed that pro-environmental intentions and PBC were the key determinants for the actual environmental behaviors of Chinese farmers. Therefore, the aforementioned observation contributes to the following hypotheses:

H1: (a) Environmental attitude, (b) subjective norms, and (c) PBC are positively related to pro-environmental intentions.

H2: (a) PBC and (b) pro-environmental intentions are positively related to PEBs.

### Religiosity and Theory of Planned Behavior

Ajzen (1991) suggested that the TPB is flexible and open to include extra variables if they are significant for the prediction and interpretation. To enhance TPB’s predictive ability, scholars have incorporated additional constructs (Karimi et al., 2021b; Karimi and Saghaleini, 2021a). Further, it was also suggested that the TPB framework could be deepened and broadened by adding new constructs or altering the path of the variables in it (Ajzen, 1991; Perugini and Bagozzi, 2001). Based on supporting evidence from the literature, the study attempts to include an additional construct in the TPB in the case of environmental behaviors, i.e., religiosity as a social background which by previous researchers has not been analyzed.

William James in his preliminary research, which was firstly published in 1902, stresses that research on religiosity provides researchers with a deeper insight into the fundamentals of human psychology (James, 1985). Religiosity determines rules, requirements, and punishments that directly affect individuals’ behavior (Harrell, 1986). Religiosity also plays a vital role in shaping the culture, values, and norms of every society (Worthington et al., 2003; Willard et al., 2016).

In the past several decades, researchers’ interest in the role of religiosity in the environment has garnered undivided attention. Since Lynn White (1967) stated that Judaism-Christianity had caused the ecological crisis due to its ethics of dominance over nature and this religion is the most anthropocentric in the world. However, other authors revealed different results concerning Christianity religiosity (Greeley, 1993; Guth et al., 1995; Slimak and Dietz, 2006; Djupe and Hunt, 2009; Jeong, 2011). They suggest that Christianity should take care of the Earth due to the “stewardship” idea which is provided in the Social Doctrine of the Church. Thus, these inconsistent results encourage the researchers to analyze the interactions between religiosity and the environment (Berry, 2013).

Monotheistic religiosity is defined as the belief in God and the commitment to act and behave in accordance with principles that are assumed to be determined by God (Weaver and Agle, 2002). It has been proven that religiosity, or one’s religious commitment, affects several personal behaviors such as PEBs (Esso and Dibb, 2004; Bhuian and Sharma, 2017; Hwang, 2018). Religiosity, as one of the major sources of human values, can drastically influence the behavioral decisions criteria or standards, especially the environmental decisions (Stark and Finke, 2000; Roccas, 2005). Environmental protection and its resources are rooted in the holy books of the chief religions of the world, including Islam (Hassan, 2014). The significance of the environment in Islam can even be revealed with a quick glimpse at the surahs and verses in the Noble Quran. Many Surahs are named after natural phenomena (e.g., dawn, honey, and star) and Allah has even sworn by some of these phenomena [e.g., “I swear by the Sun” (91:1) or “I swear by the Earth” (91:6)] (Siyavooshi et al., 2019). In fact, the literature on eco-Islam is growing with an attempt to link Islamic teachings to environmental issues (Abdelzaher and Abdelzaher, 2017). Eco-Islam explains the Islamic notion of the environment and the essence of human-environmental actions. The Islamic notion of the environment indicates that God is the owner and creator of the environment and He protects and guards it. It is a symbol of God’s existence for mankind and it is an evidence of human actions. The essence of human’s environmental actions considers the environment as something human is trusted with. It introduces the environment as a God-given gift that shall not be destroyed and it emphasizes the truth God hears, sees and knows. It also stresses all actions human takes in relation to the environment and it holds humans responsible for all of their actions in the hereafter (Rice, 2006; Abdelzaher and Abdelzaher, 2017).

As regards the environmental viewpoint of Islam, it is stressed in the Holy Quran that Allah has established a balance in the ecosystem: The excessive use of natural resources can disrupt this balance and cause environmental issues. Hence, environmental protection is one of the fundamental aspects of the Islamic faith. The Islamic strategy for environmental balance supports the timely actions taken to prevent environmental crises (Akhtar, 1996). In many verses of the Quran, environmental damage and destruction are prohibited. For instance, it is stated in verse six of Surah Al-Araf: “Do not destroy the Earth after improving it.” For the followers of Islam (i.e., Muslims), there are unwritten guidelines for protecting the earth and its resources and the Earth has to be protected as a sacred place. In Islam, humankind, as the superior being and God’s caliph on Earth, has to be friendly and responsible to the Earth’s resources. Therefore, Muslims are expected to support environmental protection measures and have environmental attitudes and behaviors (Rice, 2006; Hassan, 2014; Kalamas et al., 2014). In this regard, the study by Rice (2006) in Egypt showed that religious teachings and religiosity had a significant positive relationship with the environmental behavior of students and professors at two Egyptian universities. In their study, Wang et al. (2020) reported that religiosity positively influences the environmentalist behavioral intention of Chinese tourists. Bhuian et al. (2018) also stated that religiosity has a positive influence on the behavior of Muslim consumers. Ghazali et al. (2018) concluded that religiosity positively affects the intention of Muslim consumers to buy green products in Indonesia and Malaysia. Therefore, the following hypotheses are formulated:

H3: Religiosity is positively related to pro-environmental behavioral intention.

H4: Religiosity is positively related to PEBs.

The TPB assumes that religiosity is one of the contextual factors influencing environmental attitude, PBC, and subjective norms (Ajzen and Fishbein, 2005). As stated, religiosity is a major source of personal values (Ramasamy et al., 2010; Fry et al., 2011). For example, considering God to be a just and generous being may create corresponding values. Moreover, the divine image of humans, as equally created beings, can create moral standards such as solidarity and justice (Graafland, 2017).

Experts argue that values form the basis for attitude and can indirectly affect intention and behavior through cognitive processes. According to the TPB, personal and contextual variables may affect the three motivational variables of the model and can have a larger indirect effect on behavioral intention through them (Ajzen and Fishbein, 1980). Furthermore, according to the hierarchical value-attitude-behavior theory (Homer and Kahle, 1988), personal values indirectly affect behaviors through attitudes. In other words, values play a major role in the formation of attitude and eventually lead to certain behaviors. Hence, religiosity is expected to have an indirect effect on behavioral intention through attitude, PBC, and subjective norms. As stated, one of the key values in Islam is environmental protection. Humankind, as Allah’s caliph on Earth, is encouraged to use natural resources responsibly. Religious individuals may have a more positive attitude to environmental protection due to the environmental values introduced in Islam. The symbolic interactionism theory suggests that the degree of internalization of standards derived from the religious society is determined by the religious identity salience (Weaver and Agle, 2002). As this identity becomes more salient, the probability of the individual’s behavior is influenced by “the expectations associated with that identity” increases (Graafland, 2017). Failure to abide by the prominent religious identity probably leads to high levels of emotional distress and cognitive dissonance (Fry, 2003). Therefore, individuals with high levels of religious commitment are expected to have a positive attitude toward environmental protection. In this regard, Hassan (2015) referred to the positive influence of religiosity on the attitude of Malaysian respondents. Ghazali et al. (2018) also reported a positive association between religiosity and attitude of Muslim respondents in Malaysia and Indonesia. Therefore, the following hypothesis is suggested:

H5: Religiosity is positively related to environmental attitude.

In Fishbein and Ajzen’s (2005) model, religiosity can influence individuals’ subjective norms. Since religious values and behavioral norms of a religious society influence the attitude and behavior of other individuals in the individual’s society, they may also affect the subjective norms of people that are important in the eyes of the religious person. The likelihood of the subjective norms influencing the individual’s subjective norms in the religious society increases with an increase in the individual’s involvement in the religious society (Graafland, 2017). Therefore, the following hypothesis is presented:

H6: Religiosity is positively related to subjective norms.

Although few studies have been conducted on the association between religiosity and PBC, these studies have indicated the existence of a relationship between these two variables (Vitell et al., 2009; Kashif et al., 2017). Individuals that gain a higher religiosity score show higher levels of PBC (Walker et al., 2012). Religiosity facilitates self-control by introducing standards to the individual. Religious beliefs can also offer motivation, hope, and comfort to the individuals, which enable them to maintain their abstinence even if it is difficult (Vitell et al., 2009). Hence, it could be stated that religious individuals show high levels of self-efficacy and self-confidence in performing environmental behaviors because they exhibit these behaviors in accordance with religious guidelines that are stressed in the religion. Therefore, the following hypothesis is formulated:

H7: Religiosity has a positive effect on PBC.

**FIGURE 1:**
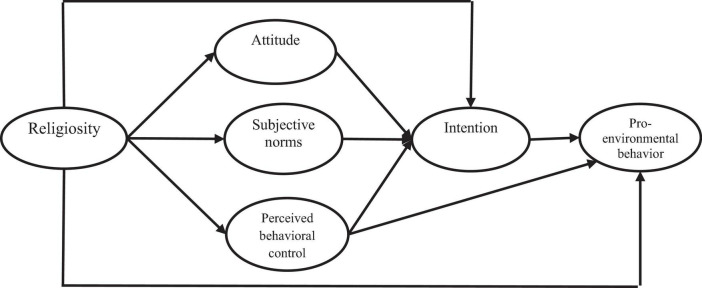
Theoretical research framework.

As mentioned previously, according to the TPB, exogenous influences or more distal factors such as religiosity can affect the behavioral intentions of individuals *indirectly* via their influences on more proximal, motivational factors such as attitudes toward behavior and PBC (Fishbein and Ajzen, 2010). In addition, the TPB supports that pro-environmental intentions should mediate the effects of motivational factors (i.e., environmental attitude, subjective norms, and perceived behavioral control) on later action, which is, pro-environmental behaviors (Ajzen, 1991). As this study hypothesized a positive association between religiosity and motivational factors; and the effect of motivational factors to pro-environmental intentions; this study investigated the mediating effect of motivational factors. Furthermore, it was hypothesized in this study that the influence of motivational factors on pro-environmental behaviors are mediated by pro-environmental intentions (Figure 1).

## Research Method

### Study Area

According to the results of Iran’s Population and Housing Census, Iran’s population was about 80 million in 2016. Muslims account for approximately 99.5% of this population, and the other religious communities (such as Christians, Zoroastrians, and Jews) constitute the remaining 0.5% (Statistical Center of Iran, 2018). The study area was Qom Province, which is situated at the center of Iran and southwest of Tehran (the capital of Iran). As one of the most religious cities in Iran, Qom is the focal point of the Shiite religion and Shiite clergymen. This county consists of 224 villages, while 110 villages have rural female facilitators. The rural female facilitators are pioneers that act as mediators between the extension agents/experts at agriculture and rural women. These women can significantly contribute to the empowerment and entrepreneurship of rural women by improving the access of rural women to the extension agricultural services and credits and by providing vocational training to them. Rural female facilitators can also play a major role in environmental protection and sustainable rural and agricultural development in rural societies by setting the scene for the participation of rural women in cultural, social, and economic activities.

### Sampling and Data Collection

The research team in the present study conducted a cross-sectional face-to-face survey of rural female facilitators in Qom Province. Data were collected over 2 weeks in April 2019. Participation in this study was voluntary and the participants were assured their information was confidential and was only used to attain the research objectives. The population of interest consisted of all rural female facilitators in Qom Province (*N* = 140). All respondents were Shiite Muslims. A total of 130 questionnaires were collected. After ruling out the incomplete and inaccurately completed questionnaires, 110 questionnaires were analyzed (response rate: 92.5%).

### Measurement

The scales used were adapted from previously validated measures after carefully analyzing the literature (Greaves et al., 2013; De Leeuw et al., 2015; Maki and Rothman, 2017; Wang et al., 2019; Ateş, 2020; Si et al., 2020). Since the scales used in the questionnaire were initially been in English, they were translated into Persian using Brislin’s (1970) procedure. Modifications and changes were made when needed to adapt the statements to the rural context and daily life of rural female facilitators. Afterward, a pilot test was conducted with 20 rural women to refine the instrument further. A few minor modifications were made based on their feedback and suggestions.

To assess PEBs, 15 questions were asked about various areas such as buying decisions, daily habits, and recycling. The respondents were asked to determine how often they performed each behavior (e.g., recycling or selling wastes such as paper, plastic, glass, and cans) in the past year (5-point scale from 1 “never,” to 5 “always”). Although self-reported past behavior is not an accurate measure of observed or actual behavior, it is often used as a proxy measure in the literature reflecting actual behavioral practices (Armitage and Conner, 2001; Bamberg et al., 2003).

Eight items were asked to assess pro-environmental intention (e.g., “I intend to recycle as much as possible in the next year”). To assess environmental attitude, five items were used (e.g., “Performing pro-environmental behaviors would be pleasant for me”), while four items were used to assess subjective norms (e.g., “My family members think that I should perform pro-environmental behaviors”). Five items were also asked to assess PBC (e.g., “I am confident in performing pro-environmental behaviors if I want to.”).

An eight-item questionnaire based on the four-dimensional religious commitment theory (Glock and Stark, 1965: belief, knowledge, experience, and ritual practice), which was designed for the Iranian culture (Karimi-Malekabadi and Esmaeilinasab, 2019), was used to assess religiosity. In this study, all the questionnaire variables, except for the demographic data and pro-environmental behaviors, were assessed based on the five-point Likert scale (from strongly disagree to strongly agree).

### Data Analysis

To analyze the research model and test the hypotheses, the structural equation modeling based on partial least squares (PLS-SEM) technique was used, with the SmartPLS 3 software (Ringle et al., 2018). PLS-SEM technique was applied due to its power in modeling under the non-normality of data and small sample size conditions. In addition, this approach allows researchers to simultaneously analyze the relationships between multi-item latent variables in a complex model (Hair et al., 2019; Manley et al., 2021). According to Hair et al. (2019), PLS-SEM is an applicable method for examining theory in development like, e.g., the extension of a given model. Moreover, the PLS-SEM has been recently intensively adopted in different research areas, including environmental behavior studies (e.g., Wang et al., 2019; Karimi et al., 2021b). Therefore, this paper employed PLS-SEM for the empirical analysis.

The two-step process suggested by Chin (2010) and Hair et al. (2019) for PLS-SEM was used to examine the hypotheses and report the results. In the first step, the measurement model was examined to assess the reliability, convergent validity, and discriminant validity of the constructs. In the second step, the structural model was examined to test the hypotheses and explanatory power of the research model.

## Results

The research results showed that 81.5% of the study women were married and 18.5% were single. Almost 90% of the study women had 1- to 4-member families. According to the research results, the average age of the study women was 41.18 years. Besides, 41.3% of the women had secondary school degrees, only 4.6% were illiterate, and more than 25% had university degrees (Table 1). Only 38.5% of the rural female facilitators participated in the activities and societies associated with the environment and the rest were not involved in any environmental activity and society. According to the results, almost 60% of the study women followed the environmental news on TV and 27.7% followed the news on the Internet. The rest of the participants also accessed this information through other means such as radio, friends, family, and experts. Table 2 provides the means, standard deviations, and correlations for the variables in the study. All variables were significantly and positively correlated with each other.

**TABLE 1:** Demographic information of respondents.

Details of respondents (*N* = 110)	Category	Frequency (s)	Percentage (%)
Age group	30<	30	27.3
	30–40	35	31.8
	40–50	33	30
	>50	12	10.9
Educational qualifications	Illiteracy	5	4.5
	Primary school	30	27.3
	High school	45	40.9
	University	30	27.3
Family size	3<	27	24.5
	3–5	46	41.8
	5–7	29	26.4
	>7	8	7.3
Marital status	Single	87	79.1
	Married	23	21.9

**TABLE 2:** Correlations, means, and standard deviation of variables in the study.

Variable	SD	M	1	2	3	4	5
1-Religiosity	3.58	0.67	-				
2-Environmental attitude	4.30	0.65	0.26*				
3-Subjective norms	4.05	0.71	0.33*	0.19*			
4-Perceived behavioral control	4.19	0.54	0.40**	0.36**	0.35**		
5-Intention	4.10	0.63	0.40**	0.45**	0.55**	0.33*	
6-Pro-environmental behaviors	4.12	0.55	0.48**	0.33**	0.41**	0.49**	0.52**

*p ≤ 0.05, **p ≤ 0.01.

### Measurement Model Assessment

To assess the measurement model, first, the reliability and validity of the measurement model were evaluated. According to Table 3, Cronbach’s alpha and composite reliability values for all constructs are higher than the acceptable level 0.7, reflecting the adequate reliability of the measurement model (Hair et al., 2019). As seen in Table 3, the average variance extracted (AVE) coefficients for all constructs are close to or higher than the acceptable level 0.5, reflecting the adequate convergent validity of the measurement model (Hair et al., 2019). Thereafter, the Heterotrait-Monotrait (HTMT) ratio was used to assess the divergent validity (Henseler et al., 2015). The HTMT ratio has to be smaller than 0.85 (Kline, 2011). As described in Table 4, all HTMT values are less than the threshold of 0.85, suggesting an adequate degree of discriminant validity of the measurement model.

**TABLE 3:** Cronbach’s alpha, CR, average variance extracted (AVE), R^2^, Q^2^ values.

Variable	α	CR	AVE	R^2^	Q^2^
1-Religiosity	0.70	0.76	0.46	–	–
2-Environmental attitude	0.77	0.85	0.59	0.07	0.03
3-Subjective norms	0.76	0.85	0.59	0.11	0.06
4-Perceived behavioral control	0.72	0.79	0.5	0.16	0.07
5-Intention	0.82	0.88	0.52	0.47	0.21
6-Pro-environmental behaviors	0.83	0.85	0.46	0.47	0.16

**TABLE 4:** Discriminant validity analysis.

Variable	HTMT				
	1	2	3	4	5
1-Religiosity	-				
2-Environmental attitude	0.36				
3-Subjective norms	0.47	0.23			
4-Perceived behavioral control	0.61	0.46	0.47		
5-Intention	0.56	0.56	0.68	0.41	
6-Pro-environmental behaviors	0.65	0.38	0.41	0.58	0.72

### Structural Model Assessment

The given relationships were analyzed using the path coefficient (β value), significance level, and effect size (*f*^2^). The explanatory and predictive abilities of the structural model were assessed using the determination coefficient (R^2^) and the predictive relevance of the model (Q^2^). Chin (1998) stated that R^2^ values above the cutoffs of 0.19, 0.33, and 0.67 were weak, moderate, and substantial, respectively, and suggested that a large R^2^ value shows a better fit of the proposed model. A Q^2^ value above 0 indicates the predictive relevance of the model with the endogenous latent variables.

According to Table 3, the R^2^ values for pro-environmental intentions and behaviors are 0.47. Considering the proposed values, these values reflect the average fit of the structural model. Based on the Q^2^ values (Table 3), the predictive power of the model for the endogenous constructs is adequate. Furthermore, as opposed to covariance-based SEM, PLS does not provide various statistical indicators for validating the theoretical models, such as χ^2^, goodness of fit index (GFI), adjusted goodness of fit index (AGFI), and other model fit measures (Henseler and Sarstedt, 2013). Tenenhaus et al. (2005) suggested a single criterion of the goodness of fit (GoF) for PLS based on the average AVE and the average R^2^. Values 0.1, 0.25, and 0.36 are described as small, medium, and large, respectively (Wetzels et al., 2009). In this study, the GoF value was 0.37 for the complete model. Therefore, it could be concluded that the research model had a relatively satisfactory overall fit and was appropriate for explaining and predicting the pro-environmental intentions and behaviors of Iranian rural female facilitators.

After assessing the fit of the measurement and structural models, and observing the relatively adequate fit of the models, the research hypotheses were analyzed and tested. The model was run by using a bootstrapping resampling procedure with 1,000 subsamples to measure the significance of the path coefficient. First, the original TPB model was tested. The original TPB model showed that environmental attitudes and subjective norms were positively related to intentions and PBC was positively related to PEBs. Next, the extended model was tested. By incorporating religiosity, the extended model improved the predictive ability of the original TPB model (The R^2^ value increased from 0.45 to 0.47 for intentions and from 0.43 to 0.47 for PEBs). In addition, it was found that the Q^2^ value of the extended model was 0.16 while the Q^2^ value of the TPB model was 0.15. Therefore, the results justified the appropriateness of incorporating religiosity into the TPB model

The significance coefficient and the standardized path coefficients for the model hypotheses are listed in Table 5. Among the key TPB components, subjective norms are the most important predictor of pro-environmental intentions (H1b: β = 0.43, *p* < 0.01). The second important predictor is environmental attitudes (H1a: β = 0.35, *p* < 0.05). PBC remains insignificant when the TPB variables and religiosity are included in the analysis. In other words, PBC is the least important factor in explaining the variance of pro-environmental intention and it does not have a significant relationship (H1c: β = −0.01, *p* > 0.05). However, the results indicate that the relationship between PBC and PEBs is significant. Therefore, hypothesis H2a is confirmed (β = 0.36, *p* < 0.01). In addition, the hypothesis about the relationship between pro-environmental intentions and PEBs is confirmed (H2b: β = 0.32, *p* < 0.01). Based on the results, religiosity does not have a significant relationship with pro-environmental intention (H3: β = 0.17, *p* > 0.05) but religiosity has a positive and significant relationship with PEBs (β = 0.21, *p* < 0.05). The results also reflect a positive and significant relationship of religiosity with attitude (H5: β = 0.26, *p* < 0.05), subjective norms (H6: β = 0.33, *p* < 0.01), and PBC (H7: β = 0.44, *p* < 0.01). To wit, a more religious individual has more PBC, higher subjective norms, and a more positive attitude toward environmental protection.

**TABLE 5:** Direct, indirect, and total effects for the sample.

Hypotheses			β	*t*-value	Supported
**Direct effect**					
Environmental attitude	→	Intention	0.35	2.57**	Yes
Subjective norms	→	Intention	0.43	4.39**	Yes
PBC	→	Intention	–0.01	0.13	No
PBC	→	PEBs	0.36	3.58**	Yes
Intention	→	PEBs	0.32	3.90**	Yes
Religiosity	→	Environmental attitude	0.26	2.46*	Yes
Religiosity	→	Subjective norms	0.33	3.09**	Yes
Religiosity	→	PBC	0.40	3.82**	Yes
Religiosity	→	Intention	0.17	1.33	No
Religiosity	→	PEBs	0.21	2.12*	Yes
**Indirect effect**					
Environmental attitude Intention	→	PEBs	0.11	2.13*	
Subjective norms Intention	→	PEBs	0.14	3.03**	
PBC Intention	→	PEBs	0.003	0.12	
Religiosity → Environmental attitude	→	Intention	0.09	2.01*	
Religiosity → Subjective norms	→	Intention	0.14	2.59**	
Religiosity → PBC	→	Intention	0.004	0.13	
Religiosity → PBC	→	PEBs	0.15	2.11**	
Religiosity → Environmental attitude Intention	→	PEBs	0.03	1.69	
Religiosity → Subjective norms → Intention		PEBs	0.14	1.99*	
Religiosity → PBC Intention	→	PEBs	–0.001	0.12	
**Total effect**					
Religiosity	→	Intention	0.40	3.54**	
Religiosity	→	PEBs	0.48	5.84**	
PBC	→	PEBs	0.36	3.69**	

*p ≤ 0.05, **p ≤ 0.01; PBC, Perceived behavioral control; PEBs, Pro-environmental behaviors.

The results also showed that religiosity was indirectly related to pro-environmental intentions via subjective norms and environmental attitudes. The results also indicated that religiosity was indirectly related to PEBs via PBC. The total effects showed that the three main predictors of pro-environmental intention are subjective norms, religiosity and environmental attitude. The total effects also indicated that religiosity, PBC and intention are the three main predictors of PEBs (Table 5).

## Discussion

The overarching goal of the present study was to identify and investigate the mechanism whereby religiosity influences the environmental intentions and behaviors of rural female facilitators in Iran by integrating religiosity into the framework of the TPB.

The results of the present study confirmed that the TPB model is a suitable framework for understanding pro-environmental intentions and behaviors of rural Muslim women in a developing country. Subjective norms and environmental attitude have a large share of the variance of rural female facilitators’ intention to perform PEBs while intention and PBC effectively predicted their PEBs. These results are in line with the results of previous studies that used the TPB model to explain behaviors (Maki and Rothman, 2017; Wang et al., 2019; Çoker and van der Linden, 2020). Therefore, the internal factor as environmental attitude and social norms is particularly important for intentions to behave in a more environmentally friendly mode. People who are interested in environmental aspects and concerned about it usually are more willing to behave in saving the environment. Among the key TPB components, subjective norms had the strongest relationship with pro-environmental intention, reflecting the substantial role of subjective norms in making decisions on rural female facilitators’ PEBs. Social pressure, particularly in collective societies, also contributes to environmentally friendly intentions. Iranians, especially rural women, are generally collectivists influenced by social norms. Hence, subjective norms are one of the main constituents of their pro-environmental intention. This finding could be mainly attributed to the strong effect of social pressures on the PEBs of rural female facilitators. If they realize that people close to them and people they respect/value (such as family members and friends) expect them to perform environment-friendly behaviors, there will be a significant change in their intention to perform PEBs. Another important finding was that despite the weak and insignificant relationship between PBC and intention, it considerably influenced PEBs, mirroring the substantial role of PBC in the exhibition and enhancement of PEBs by rural female facilitators. As stated by Ajzen (2006), PBC can serve as a representative for actual intentional control over one’s behavior. This finding highlights the importance of setting the scene for the exhibition of PEBs and eliminating any potential barrier to this path (De Leeuw et al., 2015). If rural female facilitators realize the possibility of performing PEBs and are provided with the time, opportunity, and resources required for performing the behavior and taking proper actions for environmental protection, a significant positive change will be observed in their PEBs.

In the extended TPB model, religiosity as the background of social and internal aspects was considered. The results indicated that religiosity increased the predictive power of the TPB. The findings indicated that PBC remained non-significant after the additional factor was included. Therefore, individuals were more influenced by attitude and SN than PBC. Another interesting finding from this study was that religiosity is indirectly linked to pro-environmental intention through the TPB components. These results are in line with the results from previous research (Hassan, 2015; Graafland, 2017; Kashif et al., 2017; Ghazali et al., 2018), indicating that religiosity can improve individuals’ subjective norms, PBC, and environmental attitude. It can also increase the individuals’ intention to perform PEBs through these constructs. Considering that religiosity is particularly related to morality norms which motivate people to be concern about the environment and also behave in a more environmentally friendly mode. Furthermore, religious individuals more case about other people and are more altruistic, thus it enhances the social pressure to act environmentally friendly. Therefore, the results showed that religiosity indirectly influences pro-environmental intention. This finding confirms the fundamental TPB hypothesis that suggests the TPB components can serve as mediators between personal and contextual factors and intention (Fishbein and Ajzen, 2011).

## Practical Implications, Limitations, and Avenues for Future Research

Considering the practical implications, the results of this study showed that subjective norms have the highest effect on pro-environmental intention. Hence, it is necessary to reinforce the descriptive (e.g., advertising, TV programs, and non-public environmental institutions and organizations) and injunctive norms (e.g., state laws and legal restrictions). These factors play a substantial role in the management of the rural environment (Wang et al., 2019). The Iranian society, especially Qom Province, is a religious society. This environment has a great potential for using religious values in protecting and improving the environment. As stated, social norms positively affect the individuals’ behavior, especially in rural female facilitators. Considering this capacity, religious people, communities, and meetings can refer to the emphasis Islam puts on protecting and preserving the environment in their gatherings and lectures. As a result, they can internalize PEBs in their members and fans through social norms and describe PEBs as signs of religiosity. Local policy makers and leaders can also stress religious values and direct the attention of villagers, especially women, to the emphasis religion puts on environmental protection (Siyavooshi et al., 2019). They can jointly run environmental campaigns with religious organizations to create awareness about the importance of protecting the environment. Given the effect of PBC on PEBs, authorities are recommended to make supportive policies on the facilities and financial resources for environmental protection measures. They should foster the positive environmental attitude and promote the belief among rural female facilitators in the fact that they are more capable of controlling the problems and shortages and performing PEBs by providing considerable support and facilities. Besides, raising environmental awareness by preparing and developing environmental education packages for empowering the audience, making TV documentary programs for introducing the environmental problems and risks, and holding environmental festivals, exhibitions, and conferences exclusively in rural areas can improve the TPB motivational components as well as the pro-environmental intentions and behaviors of villages, especially women.

There were also limitations on this study, which should be taken into account. Firstly, the research data was entirely collected through self-assessments. Therefore, the respondents might have overestimated their environmental behaviors to achieve social satisfaction. The previous TPB meta-analyses also confirmed that when a behavior is assessed objectively the explaining power of the model decreases as compared to the self-reported behavior (Armitage and Conner, 2001). However, it was tried to reduce the probability of bias and other variances of the common method in accordance with the suggestions by Podsakoff et al. (2003). The bias probability of the common method could not be eliminated entirely. Hence, it is recommended to assess PEBs with a more objective approach in future research. Secondly, different types of PEBs were assessed in this study. Future research can, therefore, explore a specific aspect of pro-environmental behavior and use a subjective assessment along with an objective assessment. Thirdly, this study is a cross-sectional study. As a result, the use of the structural equations modeling approach does not prove causality. Therefore, it is recommended that future research takes a longitudinal approach, which would provide a greater opportunity for analyzing causality. Fourthly, assessing actual behavior in the present study was impossible for practical reasons, therefore only self-reported past behavior was assessed as a proxy for future behavior, which does not assure a reliable measure of actual behavior and limits the interpretation of the path leading from intention to behavior in the TPB (Ajzen, 2011). Future studies might use the actual PEBs to increase measurement reliability. Finally, in this study, only the rural female facilitators in Qom Province, Iran, were studied. Therefore, more cities and even countries can be studied in future research to test the differences between the samples and different areas.

## Data Availability Statement

The raw data supporting the conclusions of this article will be made available by the authors, without undue reservation.

## Ethics Statement

Ethical review and approval was not required for the study on human participants in accordance with the local legislation and institutional requirements. Written informed consent from the participants was not required to participate in this study in accordance with the national legislation and the institutional requirements.

## Author Contributions

All authors contributed to the design and implementation of the research, to the analysis of the results, to the writing of the manuscript, and approved the submitted version.

## Conflict of Interest

The authors declare that the research was conducted in the absence of any commercial or financial relationships that could be construed as a potential conflict of interest.

## Publisher’s Note

All claims expressed in this article are solely those of the authors and do not necessarily represent those of their affiliated organizations, or those of the publisher, the editors and the reviewers. Any product that may be evaluated in this article, or claim that may be made by its manufacturer, is not guaranteed or endorsed by the publisher.
